# Intermediate Uveitis Complicated by Peripapillary Choroidal Neovascularization

**DOI:** 10.7759/cureus.31040

**Published:** 2022-11-03

**Authors:** Mohammed R Nageeb

**Affiliations:** 1 College of Medicine, Umm Al-Qura University, Makkah, SAU

**Keywords:** complications, intravitreal, peripapillary, intermediate uveitis, choroidal neovascularization

## Abstract

Choroidal neovascularization (CNV) is a very rare complication of intermediate uveitis with an incidence rate of around 2.0%. This case report examines the pathogenesis and prognosis of peripapillary CNV in a 52-year-old female patient with intermediate uveitis. The patient was initially diagnosed with intermediate uveitis and had two-month-old floaters, 20/25 vision, and snowballs in both eyes. After five years, she underwent a normal ocular checkup, which revealed peripapillary subretinal hemorrhage. The presence of a choroidal neovascular membrane was detected using fluorescein angiography and optical coherence tomography. The patient received monthly intravitreal injections of 1.25 mg of bevacizumab for four months, which improved and stabilized her eyesight, resolved the subretinal hemorrhage, and caused the CNV to recede without recurring.

## Introduction

The development of new blood vessels from the choroid that reach the retinal pigment epithelium (RPE), subretinal space, or a mix of both is known as choroidal neovascularization (CNV). It is an uncommon complication of intermediate uveitis. There have been a few studies that document such cases [1-3]. In this case report, the case of a patient with intermediate uveitis who developed peripapillary CNV is described and the pathogenesis and prognosis of this condition are examined. The patient’s neovascular membrane was considered a subsequent complication of intermediate uveitis.

## Case presentation

A 52-year-old female patient presented with floaters in both of her eyes that had been persistent for two months. The patient reported that she appeared to be seeing through a fog. The patient denied having ever experienced eye pain, redness, or sensitivity to light. A systematic review revealed no vitiligo, oral or genital sores, joint pain, or changes in bowel habits not previously reported. The patient was identified as having left-sided sensorineural hearing loss a few months prior to the presentation. There was no history of any significant medical conditions, and her family history was unremarkable. The results of the laboratory tests for the complete blood count, antinuclear antibody, and rheumatoid factor were negative. In addition, the chest X-ray was normal. The patient’s best-corrected visual acuity (BCVA) in her right eye (OD) and left eye (OS) was 20/20 and 20/25, respectively. There was 8 mmHg intraocular pressure on both sides. Anterior chambers with grade +1 anterior cells, flares, and a few small, fine keratic precipitates were seen under a slit lamp examination. No posterior synechiae were present. The results of a dilated fundus examination revealed the presence of vitreous cells and snowballs in both eyes but no snowbanking. There were no abnormalities in the retina, vessels, or optic disc. The patient was diagnosed with idiopathic intermediate uveitis and was prescribed 1% topical prednisolone as treatment. On diagnosis, the BCVA was 20/30 and 20/40 in the right and left eye, respectively. The patient was commenced on topical steroid eye drops (prednisolone) six times a day for the first week, then tapered over six weeks according to the uveitis condition on follow-ups. The uveitis responded well to treatment, and BCVA improved without any complications.

**Figure 1:**
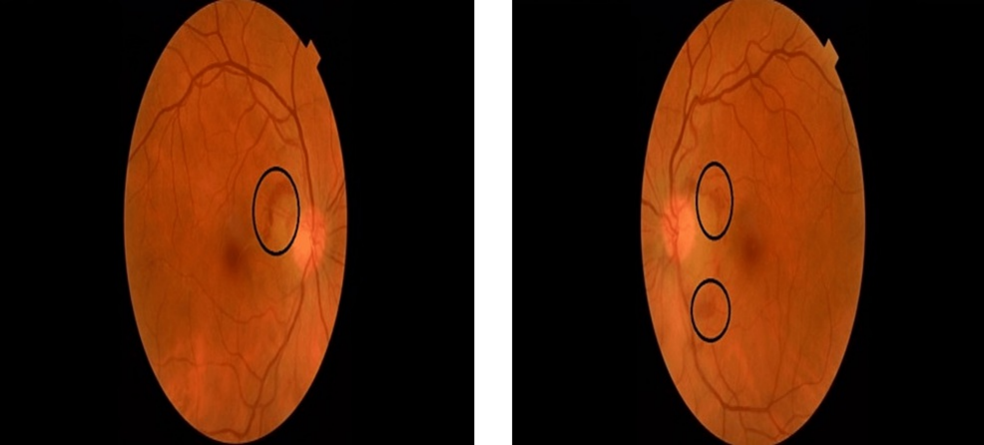
Color fundus photograph of the right and left eye. Fundus photograph showing a peripapillary subretinal hemorrhage. The picture on the left side is for the right eye and the one on the right side is for the left eye.

Five years later, she underwent a dilated fundus examination and was discovered to have a peripapillary subretinal hemorrhage in both of her eyes (Figure 1). Her BCVA was 20/25 and 20/40 in the right and left eye, respectively. She was complaining of foggy vision in the left eye.

**Figure 2:**
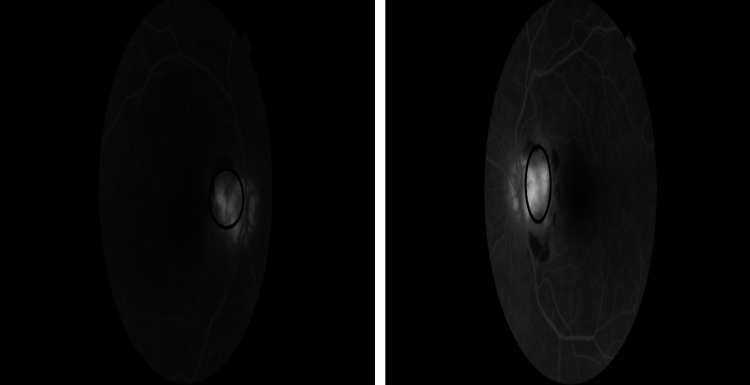
Fluorescein angiogram of the right and left eye. Fluorescein angiogram showing a choroidal neovascular membrane adjacent to the disc. The picture on the left side is for the right eye and the one on the right side is for the left eye.

Bilateral fluorescein angiograms (FA) revealed an active choroidal neovascular membrane close to the disc (Figure 2).

Monthly injections for four months of 1.25 mg of intravitreal bevacizumab were administered. The patient had a monthly check-up for eight months and is doing well, with better vision. Her eyesight is stable, and CNV had not recurred at the time of her last follow-up.

## Discussion

The aberrant growth of blood vessel complexes through the subretinal pigment epithelium or subretinal space is known as CNV [4]. It can be a complication or a complication of a variety of inflammatory conditions, such as white dot syndrome, serpiginous choroiditis, and presumed ocular histoplasmosis syndrome (POHS) [1,4,5]. The patient in this case report had no history of choroiditis or any symptoms, such as macular abnormalities or peripapillary pigmentation, that would be indicative of POHS. Optic disc drusen, congenital disc abnormalities, and tilted disc have all been linked to CNV [1,3]. The clinical characteristics of the patient and the results of the FA were unrelated to these findings. Although CNV can develop in people with papilloedema brought on by pseudotumor cerebri, the patient in this case report did not have any clinical signs of intracranial hypertension [6,7]. CNV can result from uveitis, with panuveitis or posterior uveitis being the most common types. It rarely occurs in intermediate uveitis. The literature only has a small number of examples; four of the previously reported five cases were identified to occur close to the disc (Table 1).

**Table 1:** Literature summary of cases of choroidal neovascularization in intermediate uveitis. CNV: choroidal neovascularization; NR: non-reactive (normal results)

Study	Case	Age (year)/gender	Systemic workup	Location of CNV	Active intraocular inflammation at the time of CNV	Treatment	Outcomes	Notes
Arkfeld and Brockhurstt [1]	1	29/Female	Sarcoidosis	Peripapillary	No	Argon green laser photocoagulation	Regressed CNV	Chronic disc swelling present prior to CNV
Garcia et al. [2]	2	15/Female	NR	Peripapillary	Yes	Intravitreal bevacizumab × 1	Regressed CNV	Disc swelling present
Garcia et al. [2]	3	27/Female	Idiopathic	Subfoveal	NR	None	Regressed CNV	Chronic cystoid macular edema present prior to CNV
Mehta et al. [3]	4	15/Male	Idiopathic	Peripapillary	No	Intravitreal bevacizumab × 4	Regressed CNV	Chronic disc swelling present prior to CNV
Present case	5	52/Female	Idiopathic	Peripapillary	No	Intravitreal bevacizumab × 4	Regressed CNV	No presence of disc swelling

Both focal laser and intravitreal bevacizumab were used to treat the cases, which improved their vision and caused the CNV to regress [1-3]. There are three basic growth patterns for CNV: Type 1 in which the CNV membranes are located below the RPE, Type 2 in which the growth spreads into the subretinal space, or Type 3 in which they both occur [4]. There are several pathophysiological explanations for the emergence of CNV. CNV may develop when choroidal veins pass through a possible space near Bruch’s membrane’s termination. Chronic disc edema can physically deform the peripapillary tissue, breaking the Bruch’s membrane and allowing choroidal veins to proliferate more quickly [1,3].

In previously reported cases, chronic disc edema was present prior to the beginning of CNV (Table 1). The specific pathogenesis in this case report is unknown. Clinically, the majority of CNV patients exhibit no symptoms or may exhibit metamorphopsia when the CNV membrane located in the macula causes impaired central vision and central scotoma. Because of CNV membranes, subretinal fluid or hemorrhage may develop [1,3]. Prior to starting the patient on anti-vascular endothelial growth factor (anti-VEGF) therapy, FA is largely utilized to confirm a suspected diagnosis of CNV. Both classical and occult patterns are present on FA in the CNV membrane. On FA, the classical CNV is a clearly delineated membrane that looks like a lacy web of capillary plexuses. Occult CNV, on the other hand, is the term used to designate CNV when its limits cannot be fully identified on FA and are shown as a stippled hyperfluorescence area on FA [3]. In CNV, optical coherence tomography (OCT) is primarily used to track patient response to therapy. OCT may show subretinal fluid, subretinal hemorrhage, or a hyperreflective lesion as a sign of CNV [3]. For the treatment of CNV, various therapeutic modalities exist, such as focal laser photocoagulation, photodynamic therapy (PDT), the administration of local and systemic corticosteroids, immunosuppressive therapies, and surgical excision of neovascular membranes. These therapies have limitations, are accompanied by difficulties, and have significant recurrence rates [8,9]. A patient with extensive retinal neovascularization caused by Eales disease who did not regress after sufficient photocoagulation was documented by Kucukerdonmez and colleagues [10]. VEGF is crucial for the emergence and development of CNV. Given that leukocytes and macrophages have the ability to create VEGF, which stimulates and enhances the growth of CNV [4,5], the amount of VEGF expressed in CNV that is secondary to an inflammatory condition is proportional to the number of inflammatory cells. The major goal of treating CNV is to minimize inflammation using medications that block VEGF to inhibit angiogenesis [4,5,8]. Brolucizumab, faricimab, and bevacizumab are the three intravitreal anti-VEGF medications currently in use. A complete, full-length humanized antibody called bevacizumab binds to all VEGF subtypes. Studies have demonstrated the value and potency of intravitreal bevacizumab as the initial therapy for inflammatory CNV [4,5,8,9].

## Conclusions

In this case report, peripapillary CNV in a female patient with intermediate uveitis was reported along with its pathogenesis and prognosis. Bevacizumab administered intravitreally at doses of 1.25 mg causes CNV to regress without recurrence while also stabilizing and improving the patient’s vision. A review of the literature found that peripapillary CNV in intermediate uveitis responds effectively to anti-VEGF treatment. Further research is needed to better understand the pathology of the underlying disease and the high-risk group.
